# Comprehensive Postmortem Analyses of Intestinal Microbiota Changes and Bacterial Translocation in Human Flora Associated Mice

**DOI:** 10.1371/journal.pone.0040758

**Published:** 2012-07-12

**Authors:** Markus M. Heimesaat, Silvia Boelke, André Fischer, Lea-Maxie Haag, Christoph Loddenkemper, Anja A. Kühl, Ulf B. Göbel, Stefan Bereswill

**Affiliations:** 1 Department of Microbiology and Hygiene, Charité - University Medicine Berlin, Berlin, Germany; 2 Department of Pathology/Research Center ImmunoSciences (RCIS), Charité - University Medicine Berlin, Berlin, Germany; Biological Research Centre of the Hungarian Academy of Sciences, Hungary

## Abstract

**Background:**

Postmortem microbiological examinations are performed in forensic and medical pathology for defining uncertain causes of deaths and for screening of deceased tissue donors. Interpretation of bacteriological data, however, is hampered by false-positive results due to agonal spread of microorganisms, postmortem bacterial translocation, and environmental contamination.

**Methodology/Principal Findings:**

We performed a kinetic survey of naturally occurring postmortem gut flora changes in the small and large intestines of conventional and gnotobiotic mice associated with a human microbiota (hfa) applying cultural and molecular methods. Sacrificed mice were kept under ambient conditions for up to 72 hours postmortem. Intestinal microbiota changes were most pronounced in the ileal lumen where enterobacteria and enterococci increased by 3–5 orders of magnitude in conventional and hfa mice. Interestingly, comparable intestinal overgrowth was shown in acute and chronic intestinal inflammation in mice and men. In hfa mice, ileal overgrowth with enterococci and enterobacteria started 3 and 24 hours postmortem, respectively. Strikingly, intestinal bacteria translocated to extra-intestinal compartments such as mesenteric lymphnodes, spleen, liver, kidney, and cardiac blood as early as 5 min after death. Furthermore, intestinal tissue destruction was characterized by increased numbers of apoptotic cells and neutrophils within 3 hours postmortem, whereas counts of proliferative cells as well as T- and B-lymphocytes and regulatory T-cells decreased between 3 and 12 hours postmortem.

**Conclusions/Significance:**

We conclude that kinetics of ileal overgrowth with enterobacteria and enterococci in hfa mice can be used as an indicator for compromized intestinal functionality and for more precisely defining the time point of death under defined ambient conditions. The rapid translocation of intestinal bacteria starting within a few minutes after death will help to distinguish between relevant bacteria and secondary contaminants thus providing important informations for routine applications and future studies in applied microbiology, forensic pathology, and criminal medicine.

## Introduction

Bacterial infections cause death in humans at all ages. A correct interpretation of results derived from appropriate specimens by pathologists is thus essential to finally draw the conclusion of a causal relationship between the detected pathogenic microorganism and the fatal outcome. Whereas it is standard practice to perform microbiological examinations during autopsy in perinatal and sudden, unexpected deaths in children and adults, in the vast majority of autopsies samples for microbiological examination are not taken [1]. Although a controversy about the relevance of postmortem microbiological examinations between clinical pathologists exist for decades the forensic literature, however, pretty uniquely highlights their importance [2]. Whenever a clear medical history is unavailable and autopsy or histological results are unspecific, postmortem microbiological examinations are essential tools. For instance, postmortem microbiological examinations are valuable for identification of the etiopathogenic agent in antemortal infections, for determination of the postmortem interval, for detection of food poisoning, drug-abuse related infections, and iatrogenic infections following invasive procedures, for screening of tissue donors for organ transplantation [3], [4], and for assessment of effective antemortal antibiotic treatment and infections by multi-resistant strains in the hospital setting with subsequent hygienic and legal consequences [5].

Interpretation of postmortem bacteriological results, however, are challenging in cases where potentially pathogenic microorganisms are isolated without distinct clinical and pathological signs of inflammation. Postmortal spread of bacteria from the gastrointestinal (GI) tract and thus false-positive results can occur under three major conditions: Firstly, during the agonal phase as a consequence of sustained resuscitation measures or peri-mortal ischemia at mucosal surfaces and thus compromized innate immune defences; secondly postmortem due to putrefactive growth of bacteria spreading from a focus (e.g. the GI tract) via compromized barriers to adjacent tissues or to lymphatic or blood vessels; and finally, by contamination due to improper storage of the corpse before or inadequate sampling procedures during necropsy [5], [6], [7]. An uncertain interval between the time point of death and detection of the dead body exposed to ambient temperatures and thus delayed cooling of the corpse are major unfavourable conditions for potentially false-positive microbiological postmortem results [5], [6]. Thus, knowledge of distinct quantitative and qualitative shifts of the gut microbiota over time under defined ambient conditions might help to more accurately determine the time point of death with importance for forensic pathology and criminal investigations, to judge a potentially pathogenic microorganism as the primary pathogenically relevant microorganisms as a fatal cause of death, and to reliably discriminate from putrefactive contaminants.

We and others showed that inflammatory conditions such as acute as well as chronic intestinal inflammation are accompanied by distinct changes in the intestinal microbiota composition [8], [9], [10], [11], [12], [13], [14]. Only little, however, is known so far about the gut microbiota changes in vertebrate animals postmortem under well-defined environmental conditions.

We therefore present here a systematic kinetic survey of intestinal microbiota changes and translocation frequencies to extra-intestinal tissue sites in mice under well-defined standardized conditions. After sacrifice by cervical dislocation, inbred mice with identical sex, age, and genetic background harboring a conventional or a complex human gut microbiota were kept at standard ambient conditions for up to 72 hours. At defined postmortem time points the complex microbiota of the small as well as the large intestinal lumen were analyzed applying cultural and molecular methods. In parallel, extra-intestinal organs were taken in order to assess translocation frequencies to mesenteric lymphnodes (MLNs), spleen, liver, kidney, and cardiac blood by culture. Finally, apoptotic and regenerative as well as innate and adaptive immune cells were quantified postmortem in the intestines *in situ* by immunohistochemistry. The presented results will provide important information for scientists and routine application touching interdisciplinary overlaps of applied and clinical microbiology, medical and forensic pathology, as well as criminal medicine.

## Results

### Kinetic Analysis of Changes in Colonic Microbiota Composition in Mice Harboring a Conventional Microbiota *postmortem*

**Figure 1:**
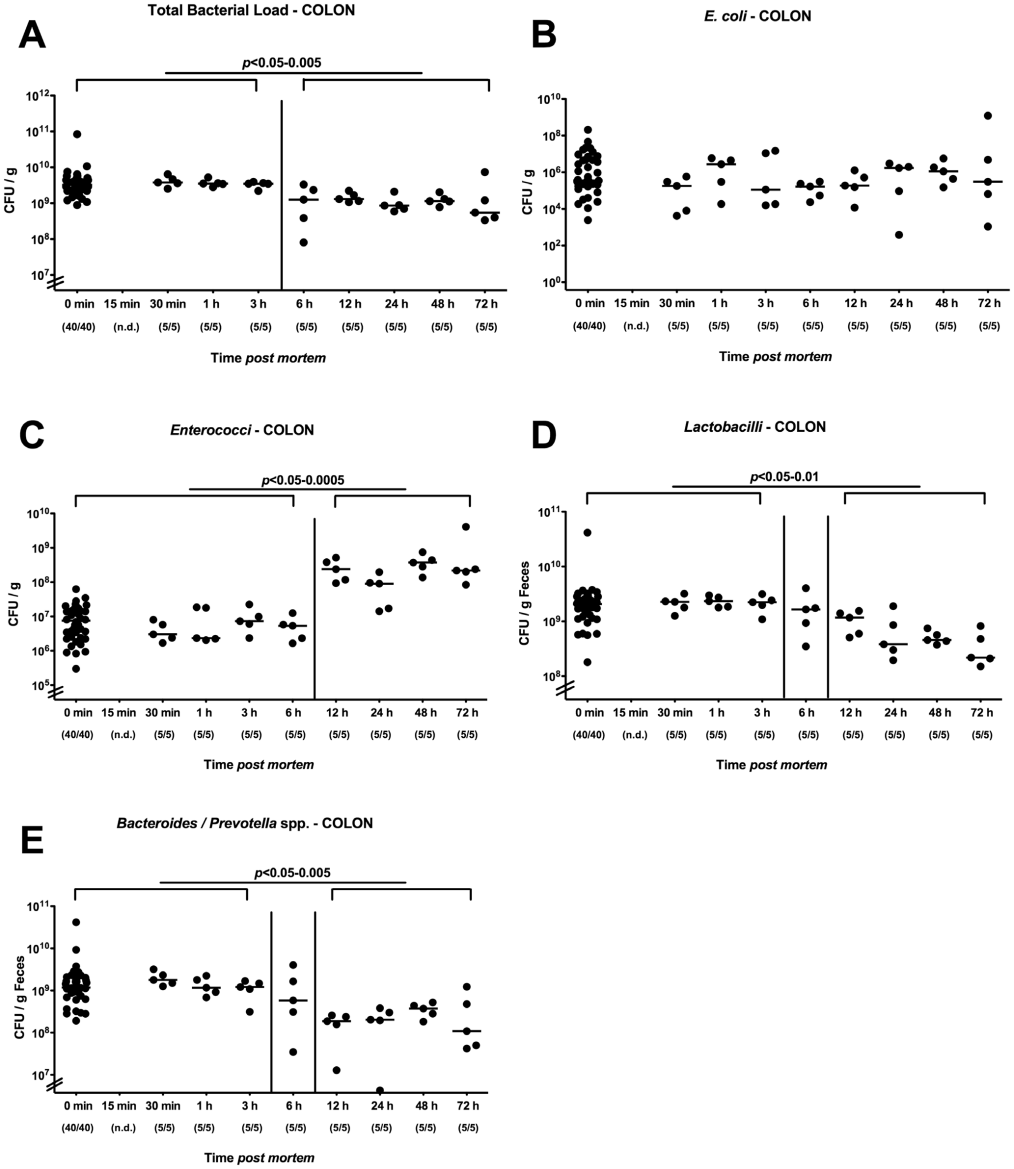
Kinetic cultural analysis of colonic microbiota changes postmortem in conventional mice. Mice harboring a conventional intestinal microbiota were sacrificed by cervical dislocation and kept at constant ambient conditions. Main gut bacterial groups of the colonic lumen contents taken at defined time points postmortem (as indicated on the x-axis) were quantified by culture (see Methods). (**A**) Total bacterial counts as well as numbers of (**B**) *E. coli,* (**C**) *Enterococci*, (**D**) *Lactobacilli,* (**E**) and *Bacteroides/Prevotella* spp. were determined by detection of colony forming units (CFU) per gram feces on appropriate culture media. Numbers of animals harboring the respective bacterial species out of total number of analyzed animals are given in parentheses. Medians and significance levels (*P-*values) determined by Mann-Whitney-U test are indicated. n.d. indicates not determined. Data shown are representative for three independent experiments.

Given that acute and chronic intestinal inflammation is accompanied by distinct gut microbiota changes, but only little is known about gut flora shifts in vertebrate animals after death under defined conditions, we performed a kinetic survey of postmortem (p.m.) changes in the main bacterial groups within the colon lumen by culture after mice harboring a conventional microbiota had been sacrificed by cervical dislocation and stored at constant ambient conditions for up to 3 days (see Methods). Between 3 and 6 hours p.m. a slight decrease of the total intraluminal bacterial loads (less than 0.5 orders of magnitude) within the colon lumen could be determined remaining at comparable levels thereafter until the end of the observation period (**Fig. 1A**). This drop in the total bacterial load was mainly due to significant decreases of the microaerophilic lactobacilli population (approximately 1.5 orders of magnitude difference between mice alive and 72 hours p.m.; Fig. 1D) and of 1 order of magnitude of the Gram-negative, obligate anaerobic *Bacteroides/Prevotella* spp. between 3 and 12 hours p.m (**Fig. 1E**). Whereas the *E. coli* counts were unchanged (**Fig. 1B**), numbers of enterococci, however, increased by approximately 2 orders of magnitude between 6 and 12 hours p.i. and remained stable until 72 hours p.i. (**Fig. 1C**).

### Kinetic Analysis of Changes in Ileal Microbiota Composition in Mice Harboring a Conventional Microbiota *postmortem*

Given that cultural methods are tidiuos, time-consuming and require special expertise for detection and subcultivation of fastidious species such as obligate anaerobic bacteria when performing a detailed survey of the complex intestinal microbiota, we applied molecular methods which not only assessed replicating bacteria but also the entire bacterial “genetic mass”, including bacteria having died in the meantime, thus contributing to a more complete picture of the complex microbiota.

**Figure 2:**
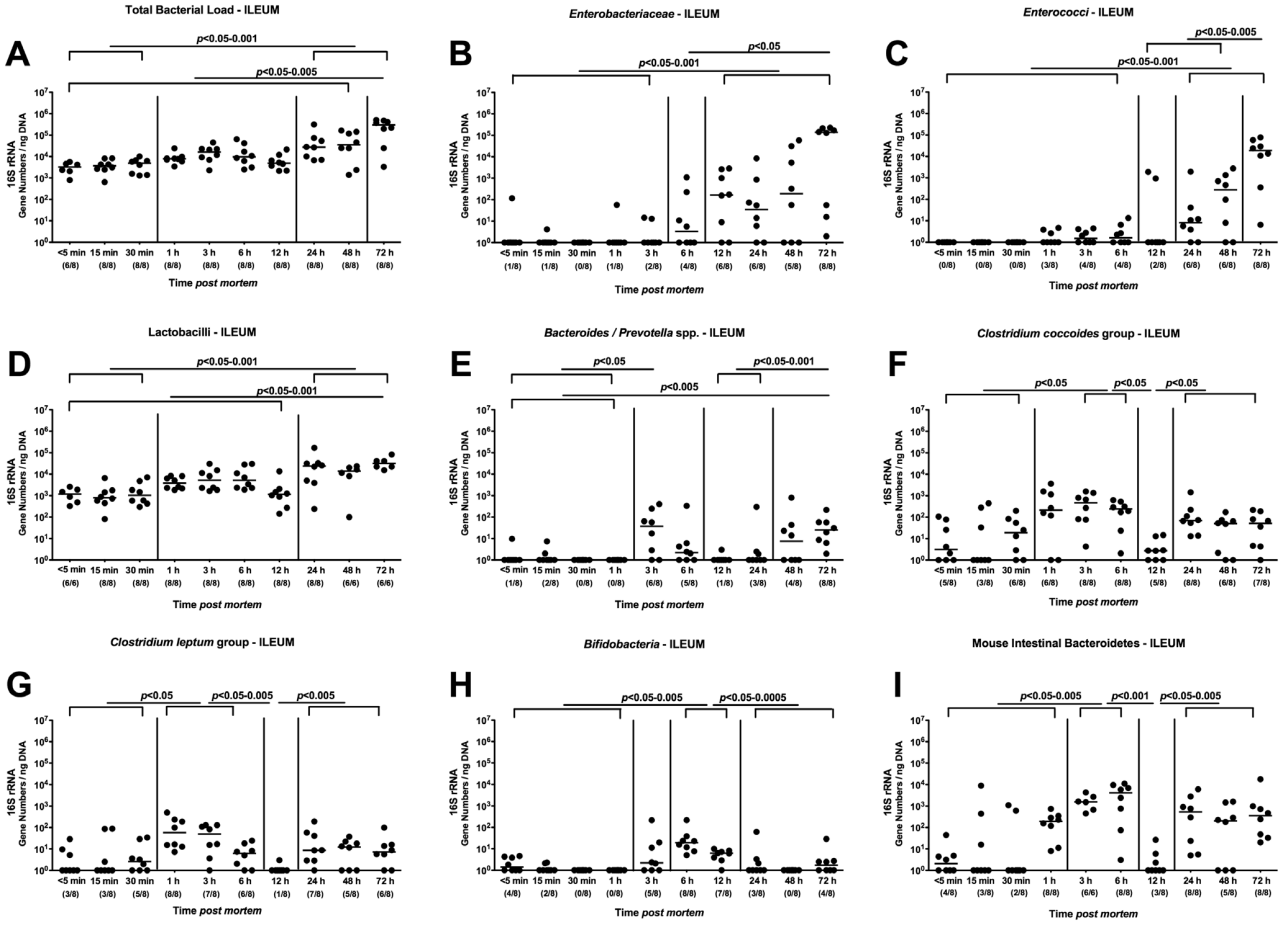
Kinetic molecular analysis of postmortem ileal microbiota changes in conventional mice. Mice harboring a conventional intestinal microbiota were sacrificed by cervical dislocation and kept at constant ambient conditions. By quantitative Real-Time-PCR analyses amplifying bacterial 16S rRNA variable regions, main gut bacterial groups were quantified in murine luminal ileum samples taken at defined time points postmortem as indicated on the x-axis (see Methods). (**A**) Total bacterial load, (**B**) *Enterobacteriaceae,* (**C**) *Enterococci,* (**D**) *Lactobacilli,* (**E**) *Bacteroides/Prevotella* spp., (**F**) *Clostridium coccoides* group, (**G**) *Clostridium leptum* group, (**H**) *Bifidobacteria*, and (**I**) Mouse Intestinal Bacteroidetes numbers were determined by detection of 16S rRNA gene numbers/ng DNA. Numbers of animals harboring the respective bacterial group out of total number of analyzed animals are given in parentheses. Medians and significance levels (*P-*values) determined by Mann-Whitney-U test are indicated. Data shown are representative for three independent experiments.

We therefore performed quantitative RT-PCR analyses by specific amplication of the 16S rRNA genes of up to 9 main bacterial groups within the intestinal microbiota thereby also reliably assessing fastidious species. Given that the large intestinal microbiota changes over time observed in our cultural pilot experiment were rather subtle (**Fig. 1**), we next focussed on the luminal microbiota within the distal part of the small intestine (terminal ileum). Whereas the total load of cultivable bacteria in the colon lumen had decreased over time postmortem, the total eubacterial load of the ileum lumen at 72 hours p.m. was significantly higher as compared to any other time points before (**Fig. 2A**). In addition, the total loads determined at 24, 48 and 72 hours p.m. were significantly higher as compared to those at 5, 15 and 30 min p.m., mainly due to higher *Enterobacteriaceae* (**Fig. 2B**), enterococci (**Fig. 2C**) and lactobacilli (**Fig. 2D**) loads in the small intestine. The enterobacteria and enterococci population significantly increased at 12 and 24 hours p.m., respectively, to maximum loads at 72 hours p.m. (**Fig. 2B, C**). Interestingly, the obligate anaerobic groups such as *Bacteroides/Prevotella* spp. (**Fig. 2E**), *Clostridium coccoides* and *leptum* groups (**Fig. 2F, G**) as well as the Mouse Intestinal Bacteroides followed a bi-phasic detection pattern: Whereas those species had significantly increased within 3 hours p.m., they declined until 12 hours back to baseline levels followed by another significant increase until 72 hours p.m. The bifidobacteria increased until 6 hours p.m. and dropped back to baseline numbers at 24 hours p.m. Taken together, molecular analyses revealed that bacterial communities within the small intestinal lumen underly distinct quantitative and dynamic shifts over time postmortem, surprisingly already within a few hours after death - most likely according to the bacterial growth behaviors and nutritional requirements.

### Bacterial Translocation Postmortem in Mice Harboring a Conventional Microbiota

In forensic medicine, identification of bacteria as a potential cause of death is essential, but correct interpretation of positive results hampered by species translocating during the peri- and/or post-mortal phase. We therefore performed a kinetic postmortem survey of culture-positive extra-intestinal tissues harboring translocated bacteria under standardized conditions, i.e. at ambient temperature to get a qualitative assessment of putrefactive changes over time.

**Figure 3:**
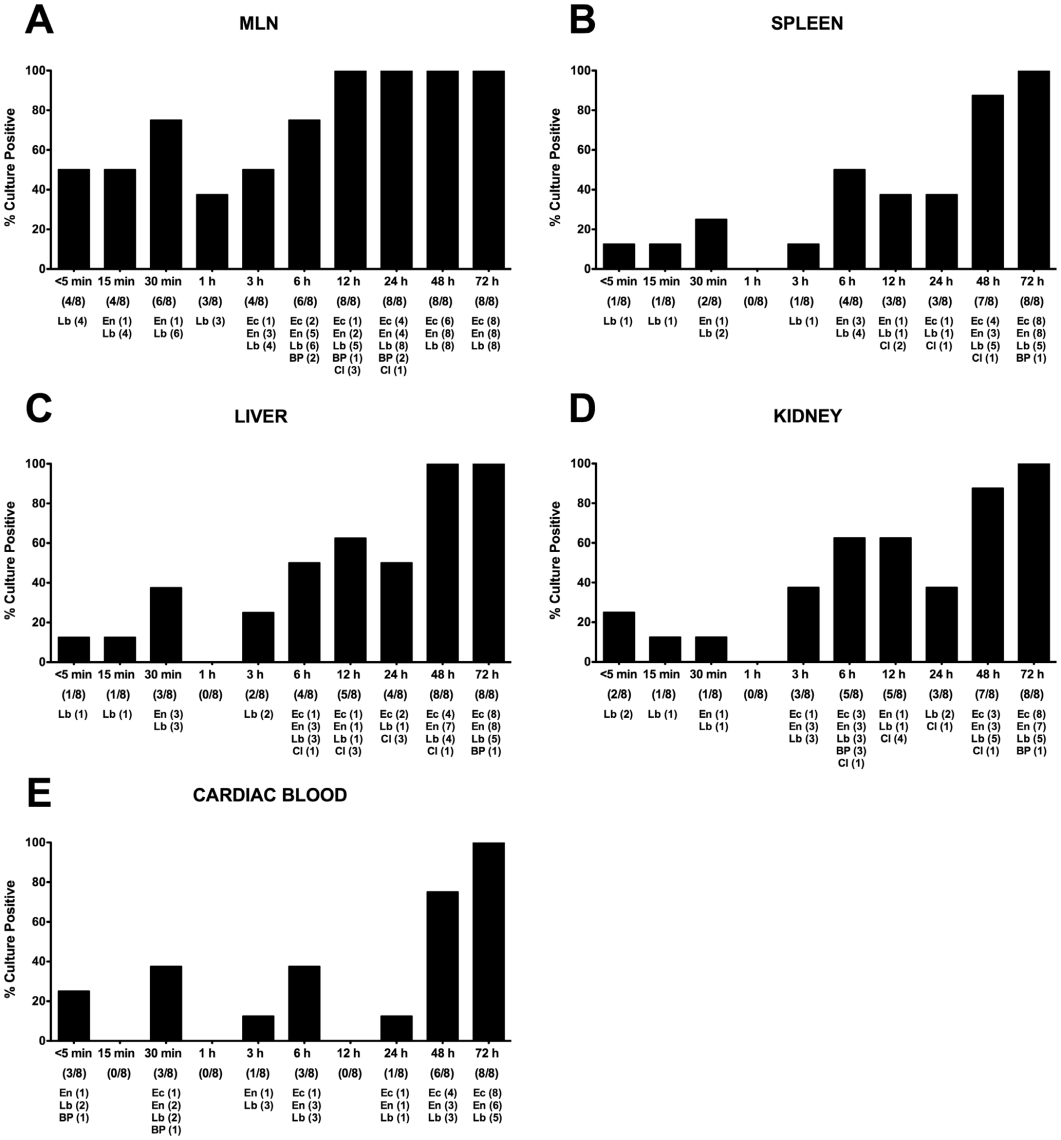
Postmortem bacterial translocation in conventional mice. Mice harboring a conventional intestinal microbiota were sacrificed by cervical dislocation and kept at constant ambient conditions. For qualitative detection of bacterial translocation at defined time points time postmortem (as indicated on the x-axis), (**A**) mesenteric lymphnodes (MLN), (**B**) spleen, (**C**) liver, (**D**) kidney, and (**E**) cardiac blood were transferred into thioglycolate enrichment broths each at defined time points (as indicated on the x-axis). Grown bacterial species such as *Lactobacillus* spp. (Lb), *Enterococcus* spp. (En), *E. coli* (Ec), *Bacteroides/Prevotella* spp. (BP), and *Clostridium* spp. (Cl) were isolated and identified as described in Methods. Bars indicate frequencies of culture positive organs (in %). Numbers of animals with culture-positive samples out of the total number of analyzed animals (as indicated below the respective postmortem time point on the x-axis) and absolute numbers of samples culture-positive with the respective bacterial species (below the latter) are given in parentheses. Data shown are representative for three independent experiments.

The highest rates of culture-positive samples were assessed in mesenteric lymph nodes (MLNs) of SPF mice. Of note, already within 5 min p.m. more than 50% of MLNs were culture-positive due to translocated lactobacilli. Until 30 min p.m. the detection rate increased up to >75% followed by a decrease to approximately 40% at 1 hours p.m., and another subsequent increase thereafter. At 12 hours, 100% of MLNs were culture-positive due to translocation of multiple bacterial species originated from the murine intestine such as *E. coli*, enterococci, *Bacteroides/Prevotella* spp., clostridia, and - with the highest detection rate - lactobacilli (**Fig. 3A**). Interestingly, at 1 h p.m., bacterial translocation rates were lowest in all investigated compartments (**Fig. 3**) with virtually no bacterial growth in spleen, liver, kidney, and blood (**Fig. 3 B–E**). Furthermore, a bi-phasic detection pattern could be observed: 15–50% of samples derived from MLNs, spleen, liver, kidney, and blood were culture-positive until 30 min p.m., followed by a decline of positivity rates at 1 h, whereas thereafter the rates of culture-positive organs increased to maximum levels of >80% at 48 and 72 hours p.m. (**Fig. 3**). Again, the most frequently detected species were lactobacilli until 3 hours p.m. Thereafter the complexity of detected species increased up to 4–5 different bacterial groups (**Fig. 3 B–D**). Strikingly, already within 5 min p.m., 25% of blood cultures were positive for enterococci, lactobacilli, and/or *Bacteroides/Prevotella* spp. (**Fig. 3E**). At 72 hours postmortem all cardiac blood samples were culture-positive for *E. coli* (100%), enteroccci (75%) and lactobacilli (62.5%).

### Postmortem Inflammatory, Regenerative and Immune Cell Responses in Ileal Mucosa of Mice Harboring a Conventional Gut Microbiota

**Figure 4:**
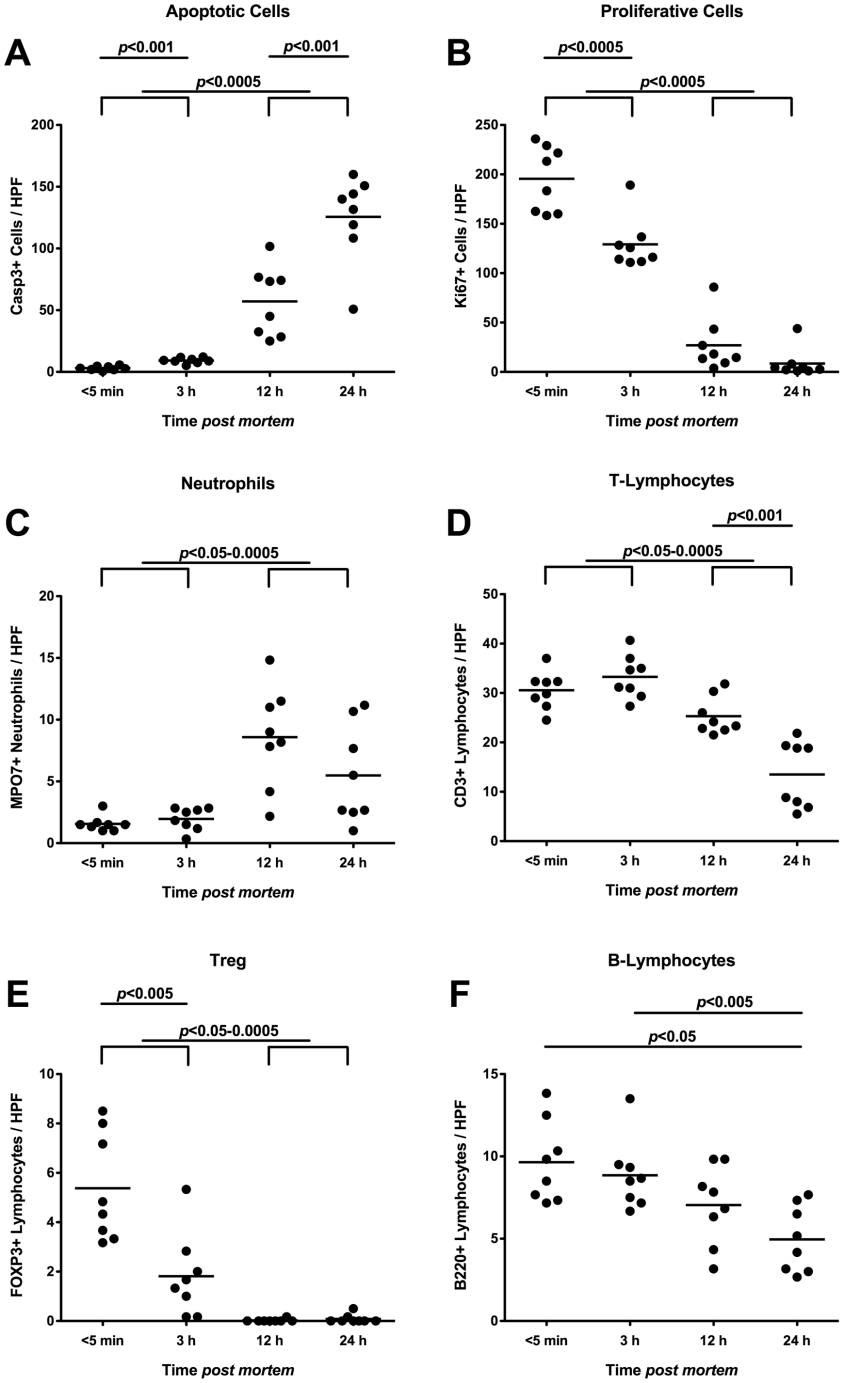
Inflammatory, proliferative and immune cells in ilea of deceased conventional mice. Mice harboring a conventional intestinal microbiota were sacrificed by cervical dislocation and kept at constant ambient conditions. Ileum biopies were taken at defined time points postmortem as indicated on the x-axis. The average numbers of (**A**) apoptotic cells (positive for caspase-3, Casp3), (**B**) proliferative cells (positive for Ki67), (**C**) neutrophilic granulocytes (Neutrophils, positive for MPO7), (**D**) T-lymphocytes (positive for CD3), (**E**) regulatory T-cells (positive for FOXP3, Treg), and (**F**) B-lymphocytes (positive for B220) from at least six high power fields (HPF, x400 magnification) per animal (n = 8) were determined microscopically in immunohistochemically stained ileal paraffin sections (see Methods). Means (black bars) and levels of significance (*P*-values) as determined by the Student’s t-test are indicated. Data shown are representative for three independent experiments.

We next quantitated numbers of apoptotic, regenerative as well as innate and adaptive immune cells within the deceases ileal mucosa at defined time points by *in situ* immunohistochemical staining of small intestinal paraffine sections (**Fig. 4**). Within 3 hours p.m., caspase3+ cells increased significantly accompanied by further multi-fold increases until 24 hours p.m. (**Fig. 4A**). These increases in apoptotic cells were inversely related to distinct decreases in Ki67+ stained cells indicating compromised proliferative/regenerative properties (**Fig. 4B**). Whereas numbers of neutrophilic granulocytes remained unchanged until 3 hours p.m., almost 4-fold higher MPO7+ cell numbers were detected at 12 and 24 hours p.m. (**Fig. 4C**). This increase in neutrophils was accompanied by decreasing T-lymphocyte and regulatory T-cell (Treg) numbers over time: 12 hours p.m. a significant decline of CD3+ cells could be observed followed by a further drop until 24 hours p.m. (**Fig. 4D**). Interestingly, FOXP3+ Tregs had dropped by more than 60% within the first 3 hours p.m., and were completely abolished by 12 hours p.m. (**Fig. 4E**). The decrease in B-lymphocyte numbers was not as distinct as compared to those observed in T-lymphocytes. Within 24 hours p.m., the B220+ numbers declined by nearly 50% (**Fig. 4F**).

### Kinetic Analysis of Changes in Ileal Microbiota Composition in Mice Harboring a Human Microbiota *postmortem*

**Figure 5:**
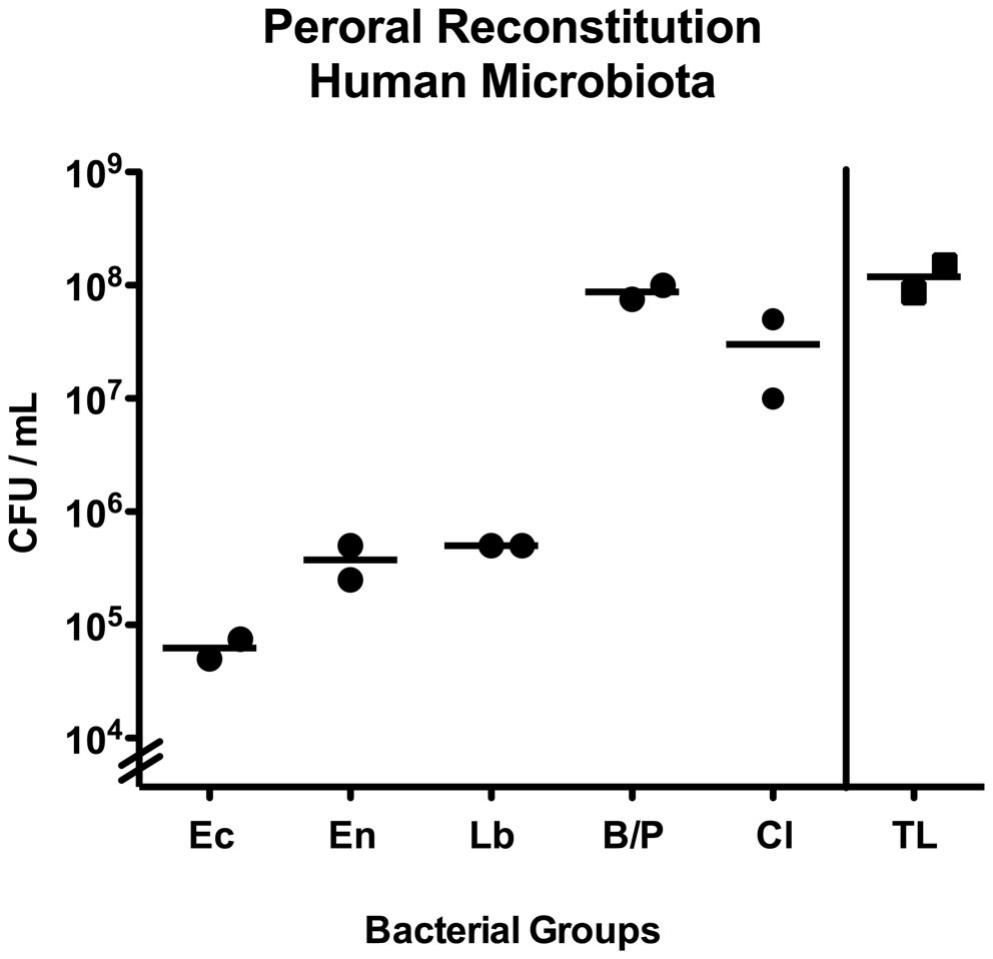
Peroral reconstitution of gnotobiotic mice with human microbiota. To generate “humanized” mice gnotobiotic animals were perorally challenged with a mixture of fecal samples derived from five individual healthy volunteers on two consecutive days by gavage as described in methods. Total bacterial loads (TL) as well as numbers of *E. coli* (Ec), *Enterococcus* spp. (En), *Lactobacillus* spp. (Lb), *Bacteroides/Prevotella* spp. (B/P), and *Clostridium* spp. (Cl) were determined by detection of colony forming units (CFU) per mL suspension on appropriate culture media.

**Figure 6:**
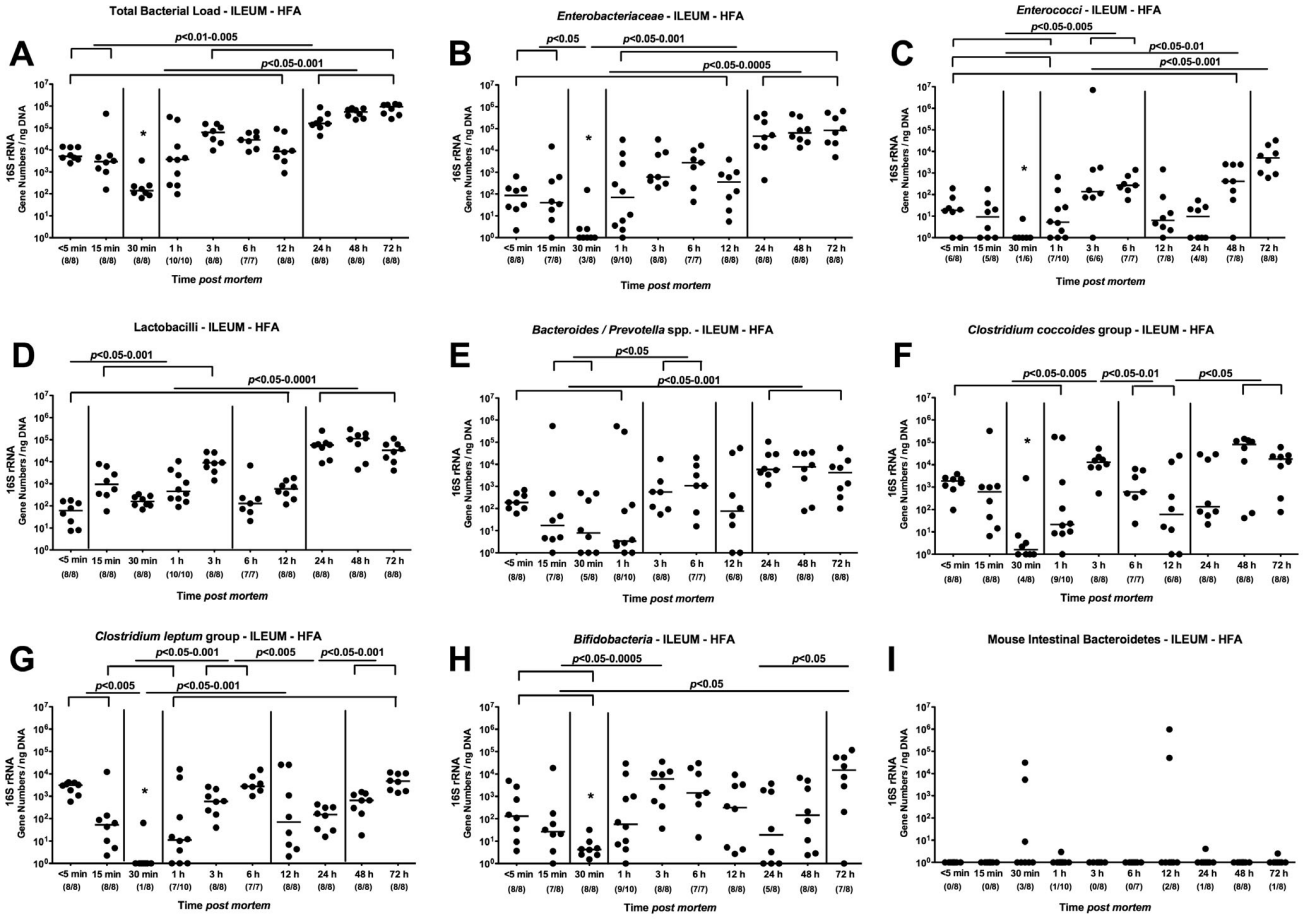
Kinetic molecular analysis of postmortem ileal microbiota changes in “humanized” mice. “Humanized” (human flora associated, HFA) mice were generated as described in Methods, sacrificed by cervical dislocation, and kept at constant ambient conditions. By quantitative Real-Time-PCR analyses amplifying bacterial 16S rRNA variable regions, main gut bacterial groups were quantified in luminal ileum samples taken from hfa mice at defined time points postmortem as indicated on the x-axis (see Methods). (A) Total bacterial load, (B) *Enterobacteriaceae,* (C) *Enterococci,* (D) *Lactobacilli,* (E) *Bacteroides/Prevotella* spp., (F) *Clostridium coccoides* group, (G) *Clostridium leptum* group, (H) *Bifidobacteria*, and (I) Mouse Intestinal Bacteroidetes were determined by detection of 16S rRNA gene numbers/ng DNA. Numbers of animals harboring the respective bacterial group out of total number of analyzed animals are given in parentheses. Medians and significance levels (*P-*values) determined by Mann-Whitney-U test are indicated. Data shown are representative for three independent experiments.

In order extend our analyses towards standardized “human-like” conditions, we next generated gnotobiotic (GB) mice by eradicating the murine microbiota with quintuple antibiotic treatment and orally replenished these mice lacking any bacteria and displaying an intact immune system with a complex human microbiota derived from healthy volunteers (see Methods). In order to generate human flora associated (hfa) mice, GB animals were challenged with a total bacterial load of 10^8^ bacteria/mL oral suspension, specifically consisting of 10^5^ CFU *E. coli*, 5×10^5^ CFU enterococci and lactobacilli, up to 10^8^ CFU *Bacteroides/Prevotella* spp. and 5×10^7^ CFU *Clostridium* spp. per mL suspension (**Fig. 5**). Cultural and molecular analyses confirmed that specific murine species such as Mouse Intestinal Bacteroides had been completely abrogated (**Fig. 6I**), whereas the applied human species stably colonized the murine GI tract (as depicted by the “<5 min data sets” of the ileal microbiota analyses in Fig. 6A–H). When comparing the dynamic changes of the murine bacterial communities postmortem with those in “humanized” animals, the shifts generally became overt at earlier time points in hfa mice. After declining until 30 min p.m., the total eubacterial loads in the ileal lumen of deceased hfa mice increased until 3 hours p.m., remaining on a comparable level until 12 hours and further increased at 24 hours p.m. (**Fig. 6A**). Interestingly, the nadir of the total load at 30 min p.m. could also be observed when quantifying enterobacteria, enterococci, clostridia and bifidobacteria, but not lactobacilli and *Bacteroides/Prevotella* spp. (**Fig. 6**). The enterobacteria loads determined between 1 hour and 72 hours p.m. were significantly higher as compared to the levels observed between <5 min and 30 min p.m. (**Fig. 6B**). Between 12 and 24 hours p.m. another significant increase of enterobacteria numbers could be measured in the ilea of hfa mice. The enterococci numbers detected at 3, 6, 48 and 72 hours p.m. were significantly higher to those determined until 1 hour p.m. (**Fig. 6C**). In addition, at the end of the observation period the enterococci loads were higher as compared to those at any previous time point of the kinetic survey (**Fig. 6C**). Interestingly, the lactobacilli numbers had already increased until 15 min. Furthermore, the lactobacilli loads determined after 24 hours p.m. were higher as compared to the numbers before 24 hours p.m. (**Fig. 6D**). Analysis of *Bacteroides/Prevotella* spp. revealed that numbers of the obligate anaerobic Gram-negative species detected between 24 and 72 hours p.m. were higher to the loads until 1 hour p.m. (**Fig. 6E**). Also for hfa mice, a bi-phasic detection pattern of the clostridia populations could be observed: After a decline until 30 min p.m., the clostridia increased until 6 hours p.m., followed by another decrease until 24 hours p.m. before increasing to maximum levels at 72 hours p.m. (**Fig. 6F, G**). The bifidobacteria population determined at the end of the survey were significantly higher as compared to the ileal loads observed in hfa mice between <5 min and 30 min p.m. (**Fig. 6H**). Importantly, analyses of the Mouse Intestinal Bacteroides revealed that the murine microbiota was absent in hfa mice (**Fig. 6I**).

### Postmortem Bacterial Translocation in Mice Harboring a Human Microbiota

**Figure 7:**
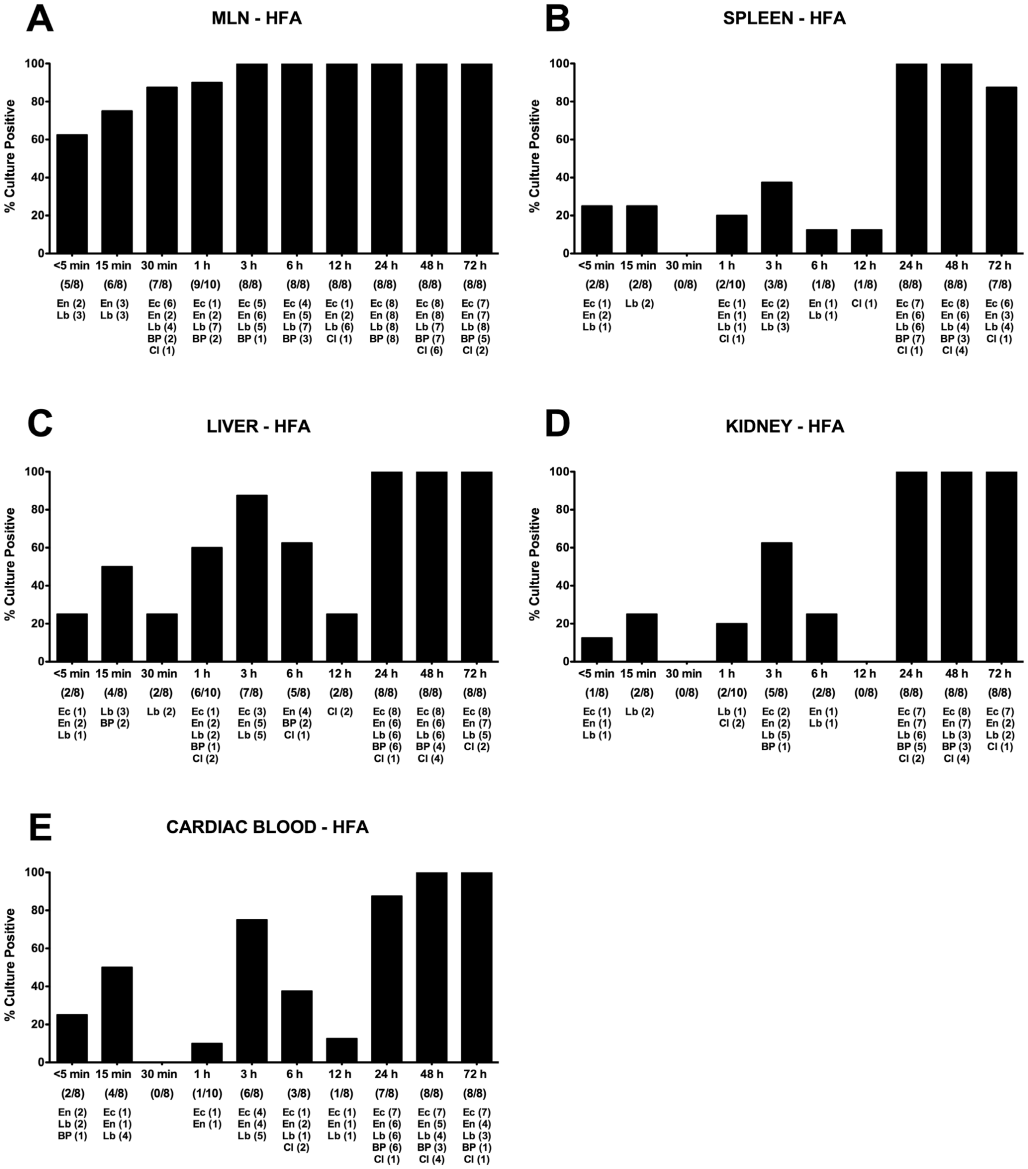
Postmortem bacterial translocation in “humanized” mice. Human flora associated (HFA) gnotobiotic mice were generated as described in Methods, sacrificed by cervical dislocation and kept at constant ambient conditions. For qualitative detection of bacterial translocation at defined time points time postmortem (as indicated on the x-axis), (**A**) mesenteric lymphnodes (MLN), (**B**) spleen, (**C**) liver, (**D**) kidney, and (**E**) cardiac blood were transferred into thioglycolate enrichment broths each at defined time points (as indicated on the x-axis). Grown bacterial species such as *Lactobacillus* spp. (Lb), *Enterococcus* spp. (En), *E. coli* (Ec), *Bacteroides/Prevotella* spp. (BP), and *Clostridium* spp. (Cl) were isolated and identified as described in Methods. Bars indicate frequencies of culture positive organs (in %). Numbers of animals with culture-positive samples out of the total number of analyzed animals (as indicated below the respective postmortem time point on the x-axis) and absolute numbers of samples culture-positive with the respective bacterial species (below the latter) are given in parentheses. Data shown are representative for three independent experiments.

We next assessed postmortem translocation of live bacteria from the intestines of deceased hfa mice to extra-intestinal tissue sites. Interestingly, in hfa mice translocated bacteria could be detected at earlier timepoints when compared to mice harboring a conventional murine microbiota (**Fig. 3** **and** **7**). In addition, more different species co-translocated to the respective compartments in hfa mice. Within 5 min p.m., more than 60% of MLNs, and as early as 3 hours p.m. virtually all MLNs were culture-positive (**Fig. 7**). Whereas until 15 min p.m. only enterococci and lactobacilli were detected, species from up to five different main bacterial groups could be cultured from MLNs thereafter. Interestingly, at 30 min p.m. spleen, kidney, and cardiac blood were completely free of bacteria in hfa mice eventhough bacteria could be detected at earlier time points (**Fig. 7B, D, E**). Also in livers of hfa mice, the culture-positivity rates were lowest at 30 min p.m. (**Fig. 7C**). These minimum bacterial translocation rates were paralleled by a decline of bacterial loads in the small intestines of hfa mice (**Fig. 6**). This nadir phenomenon of translocation rates in hfa mice had also been observed in SPF animals, but 30 min later at 1 hour p.m. (**Fig. 3B–E**). Detection rates of bacteria in spleens of hfa mice ranged between 12.5 and 25.0% until 12 hours p.m., followed by a marked increase up to 100% thereafter (**Fig. 7B**). This was also true for culture-positive livers and kidneys with detection rates between 25.0–87.5 and 0–62.5%, respectively, until 12 hours p.m. and 100% positive samples at 24, 48 and 72 hours p.m. (**Fig. 7C, D**). In 25.0%, 75.0% and 12.5% of cardiac blood samples taken at <5 min, 3 h and 12 h p.m., respectively, live bacteria could be cultured whereas after 24 hours p.m. more than 87% of blood cultures were positive with species from the five main bacterial groups originating from the intestines of hfa mice postmortem (**Fig. 7E**).

### Postmortem Inflammatory, Regenerative and Immune Cell Responses in Ileal Mucosa of Mice Harboring a Human Gut Microbiota

**Figure 8:**
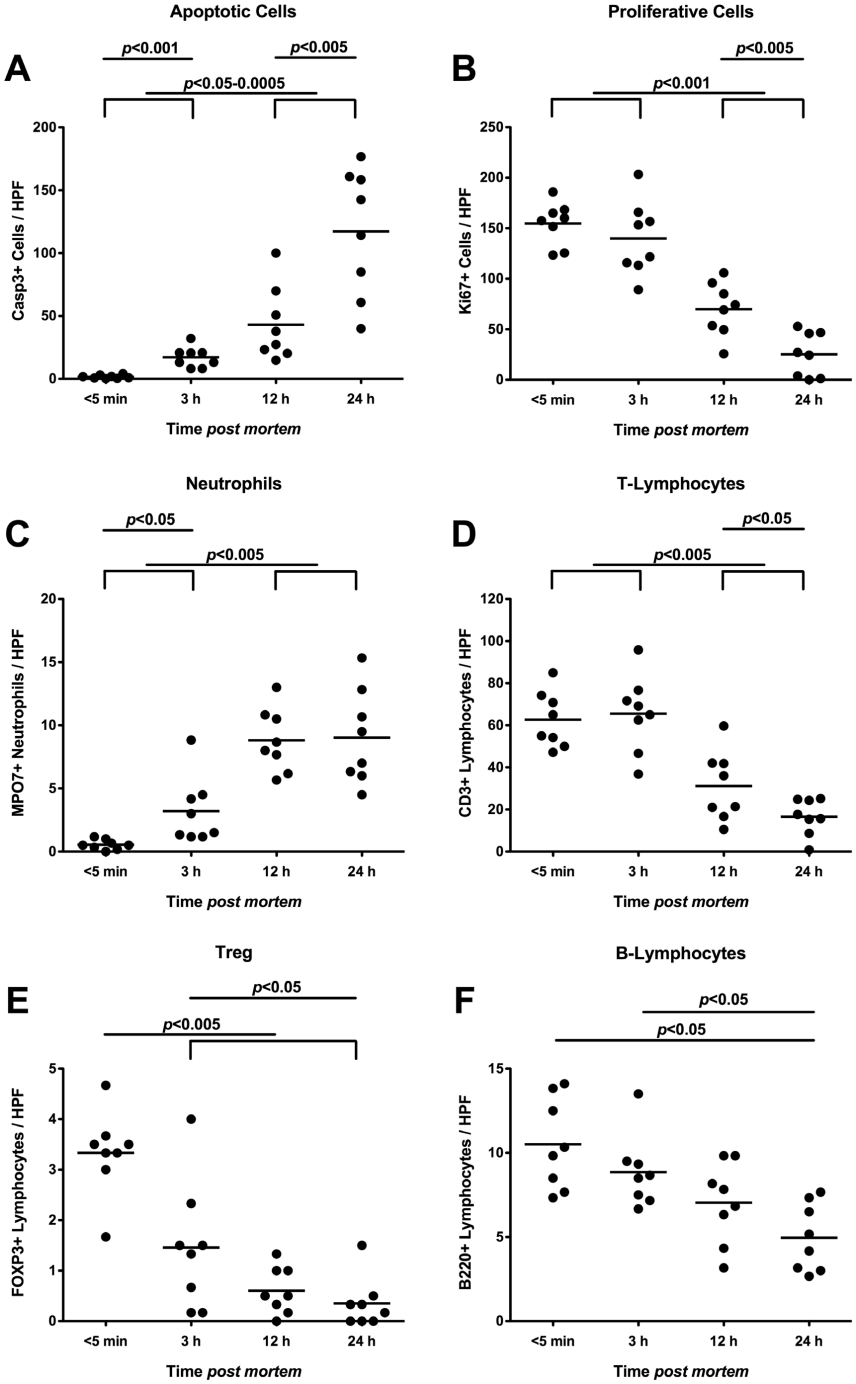
Inflammatory, proliferative and immune cells in ilea of deceased “humanized” mice. “Humanized” (human flora associated) mice were generated as described in Methods, sacrificed by cervical dislocation, and kept at constant ambient conditions. Ileum biopies were taken at defined time points postmortem as indicated on the x-axis. The average numbers of (**A**) apoptotic cells (positive for caspase-3, Casp3), (**B**) proliferative cells (positive for Ki67), (**C**) neutrophilic granulocytes (Neutrophils, positive for MPO7), (**D**) T-lymphocytes (positive for CD3), (**E**) regulatory T-cells (positive for FOXP3, Treg), and (**F**) B-lymphocytes (positive for B220) from at least six high power fields (HPF, x400 magnification) per animal (n = 8) were determined microscopically in immunohistochemically stained ileal paraffin sections. Means (black bars) and levels of significance (*P*-values) as determined by the Student’s t-test are indicated. Data shown are representative for three independent experiments.

We finally assessed apoptotic, regenerative as well as innate and adaptive immune cell responses in the distal small intestinal mucosa taken from deceased hfa mice at defined time points by *in situ* immunohistochemical stainings of ileal paraffin sections (**Fig. 8**). Just as in mice harbouring a conventional gut microbiota (**Fig. 4**), apoptotic cell numbers progressively increased multi-fold in the ilea of hfa mice until 24 hours p.m. (**Fig. 8A**), paralleled by significant decreases of Ki67+ proliferative cells between 3 h and 24 h p.m. (**Fig. 8B**). Whereas neutrophils dramatically increased p.m. reaching maximum levels at 12 h p.m. (**Fig. 8C**), T-lymphocytes progressively dropped after 3 h p.m. (**Fig. 8D**). Again, FOXP-3+ Tregs had already declined by 50% as early as 3 hours p.m. reaching minimum levels thereafter (**Fig. 8E**). Furthermore, B-lymphocyte numbers decreased between 3 and 24 hours p.m. by 50% (**Fig. 8F**) indicating that adaptive changes take place rather late in the p.m. time course.

Taken together, also in “humanized” mice distinct shifts of the complex human microbiota composition can be observed. Furthermore, p.m. translocation of live bacteria originating from the GI tract to extra-intestinal tissue sites such as MLNs, spleen, liver, kidney, and blood take place at earlier time points and more different species can be cultured as compared to SPF mice. Finally, the time course of occurring apoptotic, regenerative as well as innate and adaptive immune cells observed in deceased ileum mucosa of hfa and SPF mice are comparable.

## Discussion

### Systematic Survey of Postmortem Gut Microbiota Dynamics in Mice and Men: “Pros and Cons” of Cultural and Molecular Methods Applied in Conventional Mice

Postmortem microbiological analyses are important tools for detecting potentially infectious fatalities in cases of uncertain deaths and screening of deceased tissue donors before allogenic transplantation among others [1], [5], [6]. In cases when a detailed patient history is missing and clinical or histological results are inconclusive, for instance, reliable interpretation of obtained microbiological results is challenging, especially when judging a potentially pathogenic microorganism as primarily pathogenic relevant for fatal antemortal disease or as an irrelevant secondary contaminant [5], [6], [7]. Contamination might occur following inappropriate technical measures such as delayed cooling of the corpse or incorrect asservation of samples [5]. Furthermore, pathogenetically irrelevant bacteria might be detected following agonal spread due to intense resuscitation or other manipulative procedures, or postmortem translocation of bacterial species mostly originating from the GI tract to extra-intestinal tissue sites due to putrefactive processes at ambient temperatures [1], [5], [6], [7].

In the past, we and others showed that acute and chronic inflammation of the small and large intestinal tract is accompanied by distinct qualitative as well as quantitative changes of the luminal gut microbiota [8], [9], [10], [11]. To our knowledge, we are now the first to present a comprehensive cultural and molecular survey of gut microbiota dynamics in the small and large intestines postmortem under well-defined conditions. To accomplish this, we sacrificed mice of identical genetic background, age, and sex harboring a conventional gut microbiota by the same method (i.e. cervical dislocation), kept the corpses at ambient temperature for up to 72 hours and took luminal gut contents at defined time points. Cultural analyses of luminal colon contents revealed a significant increase of the aerobic enterococci between 6 and 12 h p.m., whereas the entire colonic bacterial loads slightly declined between 3 and 6 h p.m. due to decreases in lactobacilli and the obligate anaerobic *Bacteroides/Prevotella* spp. The observed declining numbers of obligate anaerobic species over time might be explained by the occurrence of gas pockets in the anemic organs including the gut during autolysis which in turn might exert toxic oxidative stress to obligate anaerobic bacteria.

“Classical” cultural examinations are tidiuos and require special prerequisites such as a well-equipped microbiology laboratory with technical expertise in cultivation of fastitious species such as obligate anaerobes. Given that cultivation procedures depending on bacterial growth requirements are time-consuming, a final report of results will take up to 4 to 6 days – a period of time which might be considered unacceptable in cases such as screening organ donors before allogenic organ transplantations when fast and reliable results are highly appreciated. Therefore, molecular methods come more and more into focus for routine application. Due to modern technological advances in quantitative real-time PCR amplification techniques, high through-put platforms might provide feasible future routine procedures also in forensic pathology [15], [16]. In order to determine the entire bacterial genetic mass and not to miss non-cultivable bacteria we applied RT-PCR technology and quantitatively amplified 16S rRNA of up to 9 main bacterial groups harbored by the intestinal microbiota in mice and men. Given that the large intestinal flora changes observed in conventional mice were rather subtle, we further focussed on the distal part of the small intestine (terminal ileum). The ileal total eubacterial loads were highest at the end of the observation period mostly due to maximum loads of enterobacteria, enterococci, and lactobacilli at 72 h p.m. Whereas the enterobacteria had increased between 3 and 12 h, enterococci increased between 6 and 24 h p.m. in the ileum lumen of deceased mice harboring a conventional flora.

Taken together, the applied molecular method proved suitable to reliably display the “complete picture” of postmortem intestinal flora changes at ambient conditions and revealed that between 6 and 24 hours fastly replying aerobic species displayed distinct increases in murine small intestinal lumen.

### Kinetic Patterns of Small Intestinal Microbiota Changes Postmortem in a “Humanized” Mouse Model

To mimick human postmortem conditions we generated gnotobiotic mice associated with a complex human microbiota. Following cervical dislocation, the total eubacterial load in the small intestines of “humanized” mice increased until 72 h p.m. due to significantly increasing numbers of enterobacteria, enterococci, lactobacilli, bifidobacteria and *Bacteroides/Prevotella* spp. Between 12 and 24 h p.m., a marked increase of the total eubacterial load due to higher enterobacteria and lactobacilli levels could be observed. Within 15 min p.m. lactobacilli were exclusively increasing. Surprisingly, between 15 and 30 min p.m. a marked decline of the total bacterial load could be observed due to significant decreases in enterobacteria, enterococci, bifidobacteria, and *Clostridium* spp. levels. Thereafter, however, the respective bacterial groups increased again to maximum levels at the end of the observation period. This non-linear, “undulating” detection pattern of bacterial DNA is difficult to interpret but might be due to distinct changes in the intestinal intraluminal milieu during the first 30 min as a consequence of the decreasing body core temperature and cessation of the circulation, for instance, with accumulating toxic products and shortage of nutrients and oxygen, which in turn compromizes the replication of the intestinal bacteria. Thereafter, the bacteria might adjust their metabolism and get accommodated to the new environmental conditions. Interestingly, intestinal overgrowth with enterobacteria such as *E. coli* were reported in patients suffering from inflammatory bowel diseases such as Crohn’s disease and ulcerative colitis, Graft-versus-host disease, liver injury, portal vein obstruction, prolonged enteral feeding, or under conditions of reduced bowel motility [17], [18], [19], [20], [21], [22], [23], [24], [25]. Thus, increases of intestinal enterobacteria in patients can be considered as indicator for compromized intestinal functionality.

Microbiological examinations of mucosal samples at necropsy comparing postmortem results with community control samples under defined conditions will facilitate identifying potentially invasive microorganisms [6], [26].

Taken together, the described time frames of dynamic bacterial changes in the ileum of “humanized” mice might help to more precisely determine the time point of death when a corpse was exposed to ambient conditions for an unknown period.

### Survey of Postmortem Bacterial Translocation Over Time in a “Humanized” Mouse Model

Different ways of postmortem bacterial spread have been postulated: via blood and lymphatic vessels, mucocutanoeus surfaces, and “per continuitatem” through tissues as a consequence of compromized barrier and innate immune functions [7], [27], [28], [29]. Kellerman and coworkers demonstrated *in vitro* that bacteria are capable to invade through the intact human intestinal bowel wall within 12–15 h after death [29]. *In vivo* postmortem bacterial translocation experiments were performed in rabbits and guinea pigs kept at different temperatures in the early 30 s of the last century [30], [31]. After distinct bacterial species had been introduced into the pleural cavity, *E. coli*, staphylococci and *Clostridium perfringens* could be cultured from spleen, liver, kidney, and cardiac blood between 5 and 48 h postmortem when kept at ambient temperature, whereas other commensal and pathogenic bacteria failed to exert their invasive properties [30], [31]. So far, postmortem translocation has not been investigated under well-defined ambient conditions in experimental models mimicking human flora conditions. Furthermore, only little data are available concerning bacterial translocation within the first hours p.m. [32]. In the presented study, we were surprised that as early as 5 min p.m., up to 37.5% of blood cultures taken from conventional as well as hfa mice were already culture-positive for enterococci, lactobacilli and *Bacteroides/Prevotella* spp. The intestinal bacterial growth in postmortem blood cultures in the early postmortal phase might be explained by invading intestinal bacteria during the peri-mortal phase when the local host defence is compromized due to anemia in agony [33] or by sustained resuscitation or other manipulative procedures [6], [34]. One might argue that early positivity of blood cultures in our study might be the consequence of the “traumatic” cervical dislocation procedure and the asservation technique. This explanation appears rather unlikely given that the heart was punctured under sterile conditions before opening the pleura and peritoneal cavities and in none of the animals a rupture or distortion of the GI tract as a consequence of the stretching forces during sacrifice of mice could be observed macrocopically, but minor leaks, however, cannot be entirely ruled out. Of note, polymicrobial growth in blood cultures due to agonal spread is considered to be a rare event [34]. Thus, very early polymicrobial spread via the relatively large mucosal surface of the GI tract would be a rather unexpected but so far the most reasonable explanation for the high percentage of positive blood cultures during the first 5 min postmortem. This is further supported by the high polymicrobial detection rates in mesenteric lymphnodes draining the intestinal tract: As soon as 5 min after death already 63.5% of MLNs taken from hfa mice were culture-positive with several bacterial species of intestinal origin, with progressively increasing translocation rates to up to 100% at 3 h and thereafter.

Interestingly, the positivity-rates of blood cultures taken from hfa mice did not directly correlate with the length of the postmortem interval between 15 min (50%) and 12 h (12.5%) peaking at 3 h (75%), whereas >87.5% of blood cultures were positive for up to 5 different species until 24 hours p.m. Silver and Sonnenwirth reported 31–50% of positive blood-cultures within the first 25 h p.m. [35], whereas Carpenter and Wilkins emphazised a significant postmortem translocation in the first few hours after death and observed a progressive increase of positive blood cultures from 20% at 1 h up to 40% at 18 h p.m. [28]. In a study by Saegeman and coworkers the highest rates of positive blood cultures taken from cadavers designated to organ transplantation were detected during the first 5 h p.m. (33%) proposing that rather inherent factors of the cadavers than the p.m. interval were responsible for time and extent of bacterial translocation into the blood [4]. This is well in line with a plethory of studies denying a correlation between time of necropsy and positivity of blood cultures [6], [29], [36], [37]. Of note, in the cited studies, corpses had been cooled as soon as possible upon arrival at the morgue, thereby minimizing bacterial overgrowth due to putrefaction. It is thus even more surprising that in our study where cadavers had been kept at ambient temperature which might have explained over-all higher positivity rates due to putrefactive processes, the p.m. kinetics of positive blood culture were comparable to those seen in the above cited studies. Furthermore, we could clearly show that positivity rates of blood samples were rather underlying a certain dynamic, and were neither constantly high nor progressively increasing over time within the first 24 hours. Thereafter, however, 100% of blood cultures were positive until 72 h p.m.

Given that clostridia are often detected in blood and tissues of corpses displaying putrefactive changes, it is often challenging to distinguish between ante-mortal infection and postmortem translocation [38]. In our study, clostridia could be detected as early 6 h p.m. and were abundant in 12.5 to 50% of blood cultures taken from hfa, but not conventional mice, further emphazising that our “humanized” mouse models is valuable to mimick human conditions.

Several aspects for bacterial translocation dynamics to the blood were also true for other organs. For instance, also in spleen, liver, and kidney of hfa mice translocation rates were lowest at 30 min p.m. Interestingly, at the same time point, a quantitative nadir of bacterial communities in the small intestines of “humanized” animals had been detected. It is thus tempting to speculate that a quantitative drop of the bacterial species in the intestinal lumen reduces the likelihood of bacterial translocation to extra-intestinal tissue sites via the mucosal surfaces of the GI tract under conditions of cessation of circulation and thus compromized barrier functions. Furthermore, the host-specific composition of the microbiota does rather not impact these dynamics in bacterial translocation rates given that also in conventional mice a comparable drop in translocation frequencies could be observed at 1 h p.m., eventhough 30 min later as compared to hfa mice. Thus, the time course and the frequency of translocation of intestinal bacteria to extra-intestinal tissue sites is mainly determined by postmortem changes of the intraluminal milieu in the GI tract following cessation of circulation which in turn affects the composition of the intraluminal microbiota composition.

### *In situ* Immune, Inflammatory and Proliferative Cells in Small Intestines Postmortem

In the comprehensive kinetic postmortem survey we included counts of immune, inflammatory and regenerative cells in the small intestines *in situ*. Irrespective of the host-specificity of the intestinal microbiota, apoptotic cells increased multi-fold as early as 3 h p.m., whereas cells with proliferative properties, T- and B-lymphyoctes as well as regulatory T-cells progressively declined postmortem. Whereas the regulatory T-cells were the first lymphocyte population to decline within 3 hours and to virtually completely disappear thereafter, a significant drop of B-lymphocytes was observed not earlier than 24 h p.m. Thus, innate T-cells seem to be earlier affected by postmortem conditions than humoral B-cells. Neutrophilic granulocytes, however, increased within the first 3 h p.m. remaining on constant levels after 12 h p.m. This kinetic appearance of neutrophils in the gut even postmortem is not surprising given that after circulating in the blood stream for up to 10 days, they enter the tissues, live for another 1–4 days, and can survive under anaerobic conditions due to anaerobic glycolysis [39], [40], [41]. In order to control the commensal flora and to protect from pathogens, however, oxygen is needed for generating oxidative radicals assuring proper phagocytosis function, which is progressively impaired with postmortem cessation of circulation [1], [42].

Recent observations suggested that hypoxia and reoxygenation might regulate the generation of FOXP3+ regulatory T-cells and their activity in various tissues and models [43], [44]. However, the exact mechanisms underlying distinct kinetic postmortem changes in cellular and bacterial populations await further analyis in ongoing future studies.

### Summary

Taken together, here we present comprehensive postmortem surveys under well-defined ambient conditions over time in conventional mice and mice harboring a human microbiota, which allowed for i.) dissecting microbiota changes in the small and large intestinal tract by cultural and molecular methods, ii.) investigating bacterial translocation to extra-intestinal tissue site such as mesenteric lymphnodes, spleen, liver, kidney, and blood, and iii.) analyzing apoptotic and proliferative as well as innate and adaptive immune cells in the intestines *in situ*. The microbiological results will help to i.) distinguish between primary pathogenic relevant bacteria and secondary putrefactive contaminants when screening tissue donors before allogenic transplantation or investigating the cause of uncertain deaths, ii.) to more precisely determine the time point of death and iii.) to better understand the forces driving microbiota changes under pathological conditions such as hypoxia, sepsis, and multi-organ failure. Thus, the presented study provides valuable information for future inter-disciplinary studies in the fields of medical and applied microbiology, medical and forensic pathology, and finally criminal medicine.

## Materials and Methods

### Ethics Statement

All animal experiments were conducted according to the European Guidelines for animal welfare (2010/63/EU) with approval of the commission for animal experiments headed by the “Landesamt für Gesundheit und Soziales” (LaGeSo, Berlin, Germany; registration number TVV T0114/05). Fresh human fecal samples for recolonizing gnotobiotic mice (hfa) were collected from healthy volunteers as described earlier [12]. Before sample collection written informed consent was obtained from all volunteers. Since fecal samples were obtained from co-workers of our laboratory and thus outside a clinical environment and used for re-colonization of mice only, experiments were exempted from approval by the Charité - Universitätsmedizin ethical committee according to German legacy (§15, Legal Basis for Clinical Trials).

### Mice

C57BL/6 mice were bred and maintained in the facilities of the “Forschungsinstitut für Experimentelle Medizin” (FEM, Charité - Universitätsmedizin, Berlin, Germany) under specific pathogen-free (SPF) conditions.

### Generation of Gnotobiotic Mice with a Human Gut Microbiota

To eradicate the commensal murine gut flora, mice were transferred to sterile cages and treated by adding ampicillin (1 g/L; Ratiopharm), vancomycin (500 mg/L; Cell Pharm), ciprofloxacin (200 mg/L; Bayer Vital), imipenem (250 mg/L; MSD), and metronidazole (1 g/L; Fresenius) to the drinking water *ad libitum* for 6–8 weeks as described earlier [10].

Fresh human fecal samples free of enteropathogenic bacteria, parasites, and viruses were collected from five individual healthy volunteers and animals, respectively, pooled and dissolved in an equal volume of sterile PBS, aliquoted and stored at −80°C until use. For reconstitution experiments, aliquots were thawed and bacterial communities quantified by cultural and molecular methods (refer to [10]) before gavage of mice with 0.3 mL of the “human gut flora suspension” on two consecutive days. Between independent experiments bacterial counts of groups varied of less than 0.5 log orders of magnitude.

### Sampling Procedures, Determination of Bacterial Translocation to Extra-intestinal Tissue Sites

Female 3 months old mice were sacrificed by cervical dislocation and kept at constant standardized external conditions (21°C, 50% relative humidity). Before necropsy, the murine skin/fur was thoroughly desinfected with 70% isopropanol solution for at least 30 sec in order to significantly reduce surface bacterial loads thereby minimizing the risk of contamination during section. Intestinal and extra-intestinal samples were immediately removed from each mouse within 5 minutes in parallel for microbiological, immunobiological and molecular analyses under sterile conditions at the respective time point postmortem. For qualitative detection of bacterial translocation, entire mesenteric lymphnodes, spleen, liver, kidneys, and cardiac blood were transferred into a thioglycolate broth each and incubated for maximum 7 days at 37°C. Bacterial growth was monitored daily by turbidity assessment. Aliquots from turbid broths were cultivated on solid media under aerobic, microaeropilic, and obligate anaerobic conditions and the bacterial species identified microbiologically and biochemically as described earlier [10].

### Analysis of the Intestinal Microflora

Cultural analyses, biochemical identification and molecular detection of luminal bacterial communities from luminal ileum and colon samples were performed as previously described [10], [12].

### Immunohistochemistry

Ileal and colon samples were immediately fixed in 5% formalin and embedded in paraffin. *In situ* immunohistochemical analysis of paraffin sections (5 µm) was performed as described previously [12], [45]. Primary antibodies against CD3 (#N1580, Dako, Denmark, dilution 1∶10), FOXP-3 (FJK-16s, eBioscience, 1∶100), B220 (eBioscience, San Diego, CA, USA, 1∶200), myeloperoxidase-7 (MPO-7, # A0398, Dako, 1∶10000), Ki-67 (TEC3, Dako, 1∶100), and cleaved caspase-3 (Asp175, Cell Signaling, USA, 1∶200) were used. For each animal the average number of positively stained cells within at least six representative high power fields (HPF, 400x magnification) was determined microscopically by two independent double-blinded investigators.

### Statistical Analysis

Mean values, medians, standard deviations, and levels of significance were determined using appropriate tests as indicated (two-tailed Student’s *t*-Test, Mann-Whitney-U Test). Two-sided probability (*P*) values ≤0.05 were considered significant. All experiments were repeated at least twice.
